# Sub-Inhibitory Clindamycin and Azithromycin reduce *S. aureus* Exoprotein Induced Toxicity, Inflammation, Barrier Disruption and Invasion

**DOI:** 10.3390/jcm8101617

**Published:** 2019-10-04

**Authors:** Hua Hu, Mahnaz Ramezanpour, Andrew J Hayes, Sha Liu, Alkis J Psaltis, Peter-John Wormald, Sarah Vreugde

**Affiliations:** 1 Department of Surgery- Otolaryngology, Head and Neck Surgery, University of Adelaide, Adelaide 5000, Australia; hua.hu@outlook.com (H.H.); mahnaz.ramezanpour@adelaide.edu.au (M.R.); andrew.j.hayes@adelaide.edu.au (A.J.H.); sha.liu@adelaide.edu.au (S.L.); alkis.psaltis@adelaide.edu.au (A.J.P.); peterj.wormald@adelaide.edu.au (P.-J.W.); 2 Department of Otolaryngology, Head and Neck Surgery, Shanghai General Hospital, Shanghai Jiaotong University, Shanghai 200080, China

**Keywords:** chronic rhinosinusitis, clindamycin, azithromycin, *S. aureus*, exoprotein, sub-inhibitory, mucosal barrier

## Abstract

Background: Chronic rhinosinusitis (CRS) is defined as a chronic inflammation of the nose and paranasal sinus mucosa associated with relapsing infections—particularly with *S. aureus*. Long-term treatments with protein synthesis inhibitor antibiotics have been proposed to reduce inflammation in the context chronic severe inflammatory airway pathologies, including CRS. This study assessed the effect of subinhibitory clindamycin and azithromycin on *S. aureus* exoprotein induced inflammation, toxicity and invasiveness. Methods: *S. aureus* ATCC51650 and two clinical isolates grown in planktonic and biofilm form were treated with subinhibitory clindamycin and azithromycin. Exoproteins were collected and applied to primary human nasal epithelial cells (HNECs) in monolayers and at air-liquid interface. This was followed by lactate dehydrogenase (LDH), enzyme-linked immunosorbent assay (ELISA), Transepithelial Electrical Resistance (TEER) and paracellular permeability assays to assess the effect on cell toxicity, inflammatory cytokine production and mucosal barrier structure and function, respectively. The effect of these treatments was tested as well on the *S. aureus* invasiveness of HNECs. Results: Subinhibitory clindamycin reduced *S. aureus* exoprotein production in planktonic and biofilm form, thereby blocking exoprotein-induced toxicity, reversing its detrimental effects on mucosal barrier structure and function and modulating its inflammatory properties. Sub-inhibitory azithromycin had similar effects—albeit to a lesser extent. Furthermore, clindamycin—but not azithromycin—treated *S. aureus* lost its invasive capacity of HNECs. Conclusion: Subinhibitory clindamycin and azithromycin reduce *S. aureus* exoprotein production, thereby modulating the inflammatory cascade by reducing exoprotein-induced toxicity, inflammation, mucosal barrier disruption and invasiveness.

## 1. Introduction

Chronic rhinosinusitis (CRS) is defined as a chronic inflammation of the mucosal lining of the nose and paranasal sinuses and is associated with chronic relapsing infections. It is one of the most common diseases for which antibiotics are prescribed [1]. CRS is divided into two subtypes, CRS without nasal polyps (CRSsNP) and CRS with nasal polyps (CRSwNP), based on the absence or presence of nasal polyps. Though the pathophysiological mechanisms of CRS are unclear, many studies suggest a dysbiotic microbiome associated with the pathological process [2,3,4,5,6]. *S. aureus* infections and biofilms have been associated with CRS disease recalcitrance [7] and *S. aureus* can enter the pseudostratified columnar respiratory epithelium of the sinuses, affecting the inflammatory process [8,9]. *S. aureus* secreted proteins, particularly enterotoxins, have been identified in CRSwNP mucus [10,11] and are thought to induce T-cell activation and Th2 polarisation with increased production of immunoglobulins including IgE, IgG/IgG4 and IgA [12,13]. CRS patients exhibiting *S. aureus* enterotoxin-specific IgE frequently have comorbid asthma and high mucosal Interleukin 5 (IL5) levels [14]. 

Steroids, oral antibiotics and nasal irrigations are included in the non-surgical and post-surgery treatments for CRS based on European position paper on rhinosinusitis and nasal polyps (EPOS) 2012 guidelines [15]. Macrolide antibiotics, including clarithromycin and azithromycin, are frequently used for the treatment of CRS infectious exacerbations. They act by binding to the 50S ribosomal subunit of bacteria affecting the bacterial protein synthesis [16]. Macrolides have been shown to reduce proinflammatory cytokine production and have been proposed for the long-term treatment of a number of chronic inflammatory diseases such as diffuse panbronchiolitis, bronchiectasis, CRS and cystic fibrosis [15,16,17,18]. Notably, doses that are much lower than the Minimum Inhibitory Concentration (MIC) are effective at reducing the inflammation, even when bacteria are resistant or persist in the post-treatment sputum [15,16,17,18].

Clindamycin is a classical broad-spectrum antibiotic that is used as well for the treatment of CRS exacerbations—particularly in cases of penicillin allergy or when infected with methicillin-resistant staphylococci or anaerobes. Similar to macrolide antibiotics, clindamycin binds to the 50S ribosomal subunit and inhibits the peptide chain synthesis [19]. Sub-inhibitory concentrations of clindamycin can affect *S. aureus* protein production as well [20]. Here, we tested the effect of sub-inhibitory clindamycin and azithromycin treated *S. aureus* planktonic and biofilms on the production of exoproteins and the resulting effect on attenuating their toxic, pro-inflammatory and barrier disrupting effects.

## 2. Experimental Section

### 2.1. Cells, Bacteria and Antibiotics

This study was performed in accordance with guidelines approved by the Human Research Ethics Committee of the Queen Elizabeth Hospital and the University of Adelaide. All patients gave written informed consent (reference HREC/15/TQEH/132) and all samples obtained were anonymised and coded before use. All methods were carried out in accordance with the relevant guidelines and regulations. Primary Human Nasal Epithelial Cells (HNECs) were from patients undergoing endoscopic skull base procedures without clinical or radiological evidence of sinus disease. Exclusion criteria included active smoking, age less than 18 years, pregnancy, and systemic diseases (immunosuppressive disease). *S. aureus* clinical isolates (CIs) were obtained from the sinonasal cavities of chronic rhinosinusitis patients without nasal polyps (CRSsNP; 1 with asthma (CI4) and one without asthma (CI2)). *S. aureus* ATCC51650 was obtained from the American Type Culture Collection (ATCC, Manassas, USA). Clindamycin and azithromycin were purchased from Sigma-Aldrich (St. Louis, USA).

### 2.2. Bacterial Cell Culture Supernatant Collection and Protein Concentration

A single colony of *S. aureus* ATCC51650 and CIs were cultured on 1.5% Tryptic Soy Agar (TSA) plates at 37 °C overnight. For each isolate, a 0.5 MacFarland Unit (MFU) suspension was created in 0.9% NaCl. The suspension was subsequently diluted 1:100 in Tryptic Soy Broth (TSB) and incubated for 24 h at 37 °C. Optical density at 600 nm (OD600) was tested to monitor bacterial growth. The culture was then centrifuged at 3000 g for 10 min, the supernatant collected and filtered using a 0.22 μm filter and the filtered supernatant was used in cell culture experiments. The protein concentration was measured using NanoOrange protein quantitation kit (Invitrogen, Carlsbad, CA, USA) following the manufacturer’s instructions. The bacterial pellet was resuspended in 0.9% NaCl and used for intracellular infection experiments.

### 2.3. Antibiotic Sensitivity Test

The minimum inhibitory concentration (MIC) of clindamycin and azithromycin was tested following the previously described broth microdilution method [21]. Briefly, overnight cultured bacterial colonies were collected from 1.5% TSA plates, adjusted to 0.5 MacFarland Units (MFU) with normal saline, and the bacterial suspension was 1:100 diluted in serially diluted antibiotics in 96 well plates. After a growth of 16 to 20 h at 37 °C, the MIC value was read. One, ½, ¼, ⅛ MIC of the different antibiotics were used for the bacterial treatments.

### 2.4. Primary HNECs Culture and Treatment

Primary HNECs were harvested from nasal mucosa from at least three control donors by gentle brushing in a method described by Ramezanpour et al. [22]. Extracted cells were suspended in PneumaCult^TM^- Ex Plus medium (StemCell Technologies, Vancouver, Canada). The cell suspension was depleted of macrophages using anti-CD68 (Dako, Glostrup, Denmark) coated culture dishes, and HNECs were maintained with PneumaCult^TM^-Ex Plus medium in collagen coated flasks (Thermo Scientific, Walthman, MA, USA) in a cell incubator at 37 °C with 5% CO_2_ until confluence. 5 × 10^5^ cells for each well were then transferred to collagen coated 24 well plates (Corning, New York, NY, USA) and eight well chamber slides (Corning), cultured for a further 24 h followed by application of bacterial exoproteins into the cell culture media at a concentration of 5%. HNECs were incubated for 24 h, followed by collection of the cell culture supernatant for IL6 and IL8 ELISA and Lactate Dehydrogenase (LDH) test. 

### 2.5. Air Liquid Interface (ALI) Culture and Treatment

HNECs were maintained at an Air Liquid Interface (ALI) medium, following the PneumaCult^TM^-ALI culture technique (StemCell Technologies). Briefly, Transwells (BD Biosciences, San Jose, USA) were treated with collagen (Stemcell Technologies), 7 × 10^4^ HNECs were seeded in a volume of 100 μL PneumaCult^TM^-EX plus medium into the apical chamber of Transwell plates with 500 μL of the medium in the basal chamber of each well. After three days of incubation, the apical media was disposed and 500 μL of PneumaCult^TM^-ALI medium was added into the basal chamber. The medium was changed every alternate day and HNECs at ALI (HNEC-ALI) were maintained for 21 days for development of Apical Junctional Complexes. At three weeks, the filtered *S. aureus* exoproteins from planktonic cultures were added to the apical chamber of HNEC-ALI cells at a concentration of 1:2 diluted in PneumaCult^TM^-EX plus medium to test the effect on cytotoxicity and mucosal barrier structure. 

### 2.6. Cytotoxity Assay

Cytotoxicity of HNECs was measured using an LDH release kit (Promega, Madison, WI, USA) following the manufacturer’s instructions. Briefly, 50 μL of the media from each well was transferred to a new plate, and 50 μL of LDH reagent was added to the supernatant and incubated for 30 min in the dark at room temperature (RT). 10% Triton X-100 in medium was used as positive control and 5% tryptic soy broth in medium was used as negative control. The absorbance of prepared samples was recorded at 490 nm on a FLUOstar Optima plate reader (BMG Labtech, Ortenberg, Germany), and relative viability was calculated relative to the LDH levels of negative controls (untreated cells) and positive controls. Absorbance from each well was read using a microplate reader at 490 nm. 

### 2.7. IL6 and IL8 ELISA Assay

Interleukin-6 (IL6) and Interleukin-8 (IL8) protein levels were determined with an IL6 and IL8 enzyme-linked immunosorbent assays (ELISA) kit (BD Biosciences, Franklin Lakes, NJ, USA), according to the manufacturer’s instructions. All measurements were performed in duplicate. Poly (I:C) Low Molecular Weight (LMW) (Invivogen, San Diego, CA, USA) was added at a concentration of 10 μg/mL to induce inflammation and used as positive control [23,24]. The optical density (OD) was measured at 450 nm and protein content determined using the standard curve prepared for each assay. 

### 2.8. SDS Page

After 24 h culture, 15 μL of filtered bacterial culture supernatant was run on a NuPAGE 4% to 12% Bis-Tris SDS gel (Invitrogen, Carlsbad, CA, USA) for 50 min at 200 volts. The gel was stained with colloidal blue staining kit (Invitrogen, California, USA) according to the manufacturer’s instructions and imaged on Gel Doc EZ system (Bio-Rad Hercules, CA, USA). Images were analyzed with image lab software (Bio-Rad).

### 2.9. Biofilm Culture, Treatment and Minimum Biofilm Eradication Concentration (MBEC) Assay

A 1.0 MFU *S. aureus* culture suspended in TSB was added to 96-well Biofilm inoculators (Innovotech, Edmonton, Canada). Following the manual, plates were incubated for 48 h at 37 °C on an orbital shaker at 110 rpm to allow biofilm formation. The pegs were subsequently washed with phosphate buffered saline (PBS) and dose ranges of clindamycin (50, 20, 10, 5, 2, 1, 0.5, 0.2 and 0.1 μg/mL) and azithromycin (500, 200, 100, 50, 20, 10, 5, 2 and 1 μg/mL) were added into the wells. Pegs were treated for 24 h at 37 °C with gentle shaking. After antibiotic challenge, the pegs were washed and sonicated on high for 30 min to dislodge the biofilm. The recovery plates with collected biofilm were incubated for 24 h at 37 °C to determine the MBEC (the minimum concentration of antibiotic that eradicates the biofilm). Concurrently, the AlamarBlue (Invitrogen) viability assay was then used as previously described to determine biofilm viability and anti-biofilm activity of the treatment [25]. Fluorescence was measured in a microplate reader (FLUOstar Optima, BMG Labtech) and percent reduction of biofilm after treatment calculated.

### 2.10. S. aureus Biofilm Protein Collection and Protein Concentration

A measurement of 0.5 MFU of overnight-cultured *S. aureus* isolates were diluted 1:100 and cultured in TSB medium in 50 mL Falcon tube for 48 h to form biofilms. Then, the media and bacteria were disposed, and the biofilms were washed with PBS twice gently, followed by adding fresh TSB with ½ MIC and 1 MIC clindamycin and azithromycin. After 48 h incubation, the media was disposed and biofilms were washed with PBS, followed by collection of the biofilm after sonication for 30 min and centrifugation at 4000 rcf for 10 min. The collected samples were diluted in sterile milliQ water. The protein content was tested using NanoOrange protein quantitation kit (Invitrogen) following the manufacturer’s instructions.

### 2.11. Transepithelial Electrical Resistance (TEER)

Transepithelial electrical resistance (TEER) was measured by using an EVOM volt-ohmmeter (World Precision Instruments, Sarasota, FL, USA) after adding 100 µL of PBS to the apical chamber of HNEC-ALI cultures. Only readings more than 800 Ω/cm^2^ were used for the treatments. PneumaCult^TM^-Ex Plus medium and 2% Triton X-100 in medium were used as negative and positive control, respectively, and TEER readings were carried out at 0 h, 15 and 30 min.

### 2.12. Permeability Assay

FITC-dextran 4kDa (Sigma-Aldrich, Saint Louis, CA, USA) was used to measure the paracellular permeability of HNEC-ALI. After 30 min of TEER measurement, the media in the apical chamber was changed with 3 mg/mL FITC-dextran in PneumaCult^TM^-Ex Plus medium. After 2 h of incubation at 37 °C, 40 μL samples from the basolateral compartment chamber of the Transwell was collected and transferred to 96-well plates (Corning, Cambridge, UK), and the fluorescence was measured using a microplate fluorometer (FLUOstar Optima, BMG Labtech). Relative permeability of treatments was expressed as treatment fluorescence/medium control fluorescence.

### 2.13. Immunofluorescence Staining

HNEC-ALI were fixed with 2.5% formaldehyde, permeabilized with 0.1% Triton X-100 in PBS for 10 min and blocked with serum-free blocker for 1 h (Dako, Glostrup, Denmark). HNEC-ALI was incubated with 1:100 dilution of mouse monoclonal anti-human Zonula occludens-1 (ZO-1) antibody (Invitrogen) at 4 °C overnight. After washing with TBST three times, 1:200 diluted anti-mouse Alexa Fluor-488 conjugated secondary antibody (Jackson Immunoresearch Laboratories, West Grove, PA, USA) was added and incubated 1 h at RT followed with DAPI (Sigma-Aldrich, St. Louis, USA) staining for 15 min. Then, membranes were transferred to a glass slide and a drop of anti-fade mounting medium (Dako, Glostrup, Denmark) was added before cover slipping. Samples were visualized by using LSM700 confocal laser scanning microscope (Carl-Zeiss, Oberkochen, Germany).

### 2.14. Intracellular S. aureus Infection

The bacterial pellet from the bacterial cell culture supernatant collection protocol was resuspended to 3.0 McFarland unit in 0.9% NaCl, diluted 1:12.5 in PneumaCult^TM^-Ex Plus medium and co-cultured with HNECs for 3 h (37 °C, 5% CO_2_). To remove extracellular bacteria after treatment, the cells were washed with PBS three times, treated with 4 μg/mL lysostaphin (Sigma-Aldrich, St. Louis, MO, USA) for 30 min and washed with PBS a further three times. HNECs were then lysed with sterile milliQ water for 30 min and lysates 1:10 serially diluted before plating on 1.5% TSA plates. Plates were incubated for 24 h at 37 °C to allow growth before colonies were enumerated and colony forming units per mL (CFU/mL) were determined. Concurrently, HNECs grown on chamber slides were fixed with purified methanol for 7 min at RT and Giemsa stained to assess the bacterial infection status.

### 2.15. Statistical Analysis

Microsoft Excel 2010 (Microsoft Corp, Redmond, WA, USA) and SPSS v.22 (IBM Corp, Armonk, NY, USA) were used for statistical analysis. Half MIC antibiotics and no-antibiotic bacterial supernatant treatments were compared using t-test; multi-MIC antibiotics treatments were analyzed using analysis of variance (ANOVA) followed by Least Significant Difference (LSD) post hoc comparison. All treatments were repeated three times separately using cells from three donors. Data is presented as mean ± standard error of the mean (SEM). *p* < 0.05 was considered as statistically significant.

## 3. Results

### 3.1. S. aureus MIC and Bacterial Growth with Subinhibitory Clindamycin and Azithromycin

**Figure 1:**
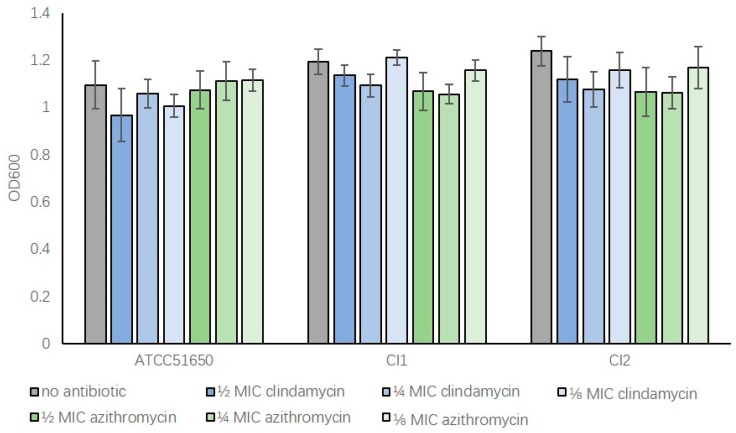
Sub-inhibitory concentrations of clindamycin and azithromycin do not significantly affect bacterial growth following 24 h incubation. The OD600 absorbance values of *S. aureus* after 24 h culture. ATCC51650, CI1, CI2 were grown without (no antibiotic, grey) or with ½, ¼, ⅛ MIC clindamycin (blue) or azithromycin (green) (*p* > 0.05). *n* = 3, bars represent standard error of means.

To determine the effect of sub-inhibitory concentrations of clindamycin and azithromycin on bacterial growth, the MIC’s of *S. aureus* ATCC 51650 and two *S. aureus* clinical isolates were first determined (Table 1). Bacteria were then treated with ½, ¼ and ⅛ MIC antibiotics for 24 h before measuring OD600 absorbance values. Compared to non-treated samples, bacteria treated with sub-inhibitory clindamycin or azithromycin had similar OD600 values indicating low antibiotic concentrations, which did not significantly affect the growth of those bacteria (Figure 1, *p* > 0.05). This allowed for a more direct comparison of antibiotic treated and untreated exoproteins in subsequent experiments without confounding issues of variable bacterial density.

### 3.2. Sub-Inhibitory Clindamycin and Azithromycin Reduced S. aureus Exoprotein Secretion 

**Figure 2:**
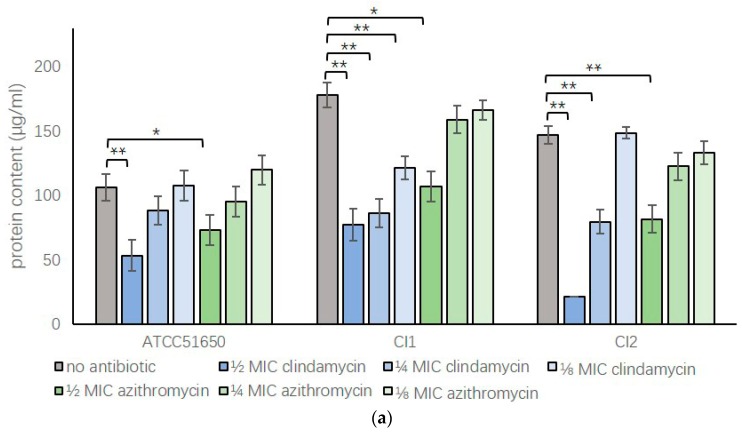

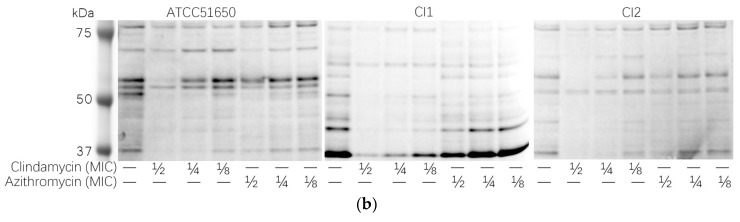
Protein content of *S. aureus* exoproteins after sub-inhibitory clindamycin and azithromycin treatment. (**a**) Protein content measured by NanoOrange (μg/mL) or (**b**) gel electrophoresis of ATCC 51650, CI1 and CI2 24-h planktonic cultures in the absence (no antibiotic, grey bar) or presence of ½, ¼ or ⅛ MIC clindamycin (blue bars) or azithromycin (green bars). *n* = 3. * *p* < 0.05, ** *p* < 0.01, one-way ANOVA followed LSD post hoc comparison.

The effect of sub-inhibitory clindamycin and azithromycin *on S. aureus* exoprotein production was assessed measuring protein concentrations and using SDS-page of the bacterial culture exoproteins. Sub-inhibitory clindamycin and azithromycin consistently reduced *S. aureus* exoprotein production in a dose-dependent way for all three isolates tested; however, the effect of clindamycin was more pronounced compared to azithromycin (Figure 2 and Table 2). 

### 3.3. Sub-Inhibitory Clindamycin Reduced S. aureus Biofilm Protein Content

**Figure 3:**
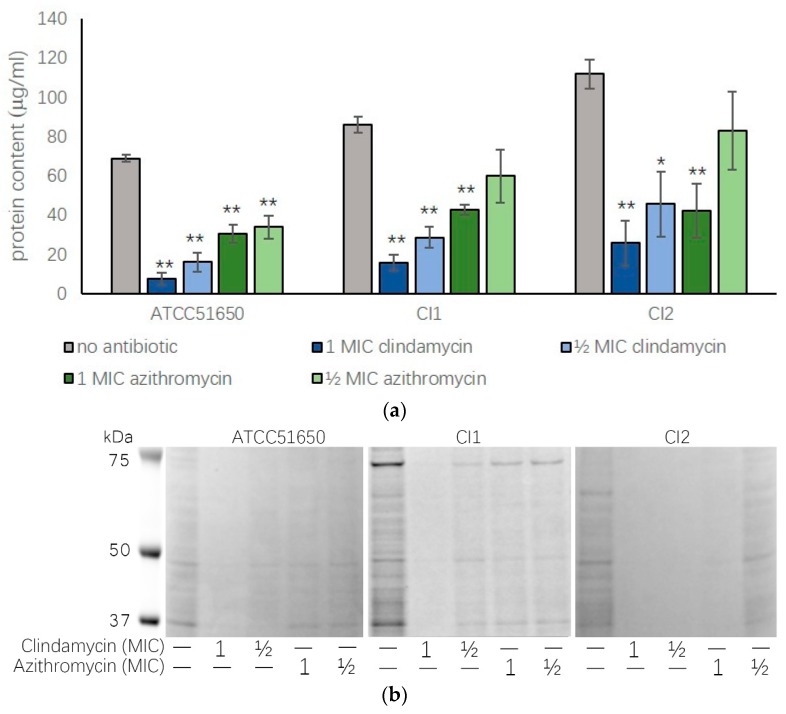
Biofilm protein content after ½ MIC and 1 MIC clindamycin and azithromycin treatment. (**a**) Protein content measured by NanoOrange (μg/mL) or (**b**) gel electrophoresis of 48-h biofilms of ATCC 51650, CI1 or CI2 in the absence (no antibiotic, grey bar) or presence of ½ MIC and 1 MIC clindamycin (blue bars) or azithromycin (green bars). (* *p* < 0.05, ** *p* < 0.01, one-way ANOVA followed LSD post hoc comparison, *n* = 3).

As *S. aureus* often forms persistent biofilm states with reduced sensitivity to antibiotics, we wanted to assess whether sub-inhibitory antibiotics had a similar effect on biofilms. We first tested the clindamycin and azithromycin minimal biofilm eradiation concentration (MBEC) for ATCC 51650, CI1 and CI2. The clindamycin (MBEC >50 μg/mL) and azithromycin (MBEC >500 μg/mL) MBEC were more than 250-fold times higher than the MIC. We then treated biofilms of ATCC 51650, CI1 and CI2 with ½ MIC and 1 MIC of clindamycin and azithromycin for 48 h and collected and quantified the biofilm proteins. Results showed ½ MIC and 1 MIC clindamycin and 1 MIC azithromycin significantly reduced biofilm protein content (*p* < 0.05) (Figure 3 and Table 3).

### 3.4. Sub-Inhibitory Clindamycin Reduced Bacterial Exoprotein-Induced Cytotoxicity of HNECs

**Figure 4:**
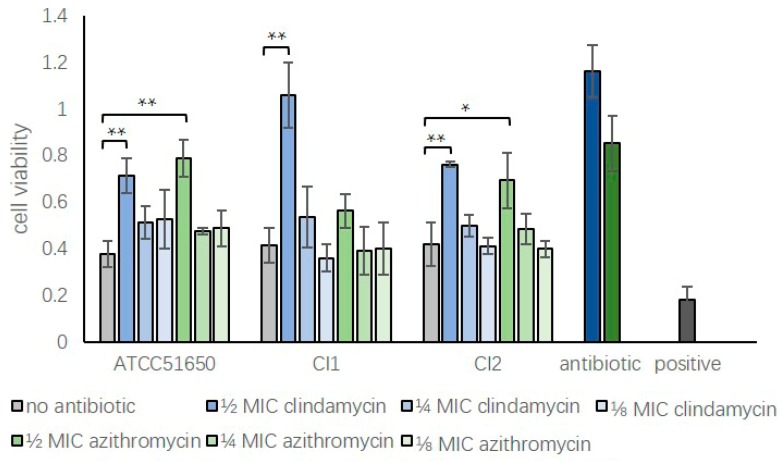
Reduction of S. aureus exoprotein-induced cytotoxicity of HNECs by treatment with sub-inhibitory clindamycin or azithromycin. Cell viability relative to negative control (5% tryptic soy broth in cell culture medium) of HNECs treated for 24 h with untreated *S. aureus* (ATCC 51650, CI1 and CI2) exoproteins (no antibiotic, grey bars) or ½, ¼ or ⅛ MIC clindamycin (blue bars) or azithromycin (green bars) treated exoproteins. Positive control = 10% Triton X-100; Antibiotic control = ½ MIC clindamycin (dark blue) or azithromycin (dark green) in medium. *n* = 3; * *p* < 0.05, ** *p* < 0.01, one-way ANOVA, followed by LSD post hoc comparison).

Exoproteins from the three planktonic *S. aureus* strains left untreated or treated with sub-inhibitory clindamycin or azithromycin for 24 h were applied to HNECs for 24 h, followed by measuring cell viability using LDH assays. Compared with negative control (5% tryptic soy broth in cell culture medium), the addition of non-treated exoproteins (5% in cell culture medium) from *S. aureus* ATCC 51650 and two clinical isolates significantly reduced the cell viability to 37.67 ± 5.44% for ATCC 51650, 41.43 ± 7.50% for CI1 and 41.86 ± 9.36% for CI2 (*p* < 0.05). Half MIC clindamycin or azithromycin reduced the *S. aureus* exoprotein-induced HNEC toxicity for 3/3 and 2/3 isolates, respectively (*p* < 0.05). Direct application of ½ MIC antibiotics at respective concentrations did not affect cell viability compared to negative control (Figure 4).

### 3.5. Sub-Inhibitory Clindamycin Reversed S. aureus Exoprotein Induced IL6 And IL8 Secretion by HNECs

**Figure 5:**
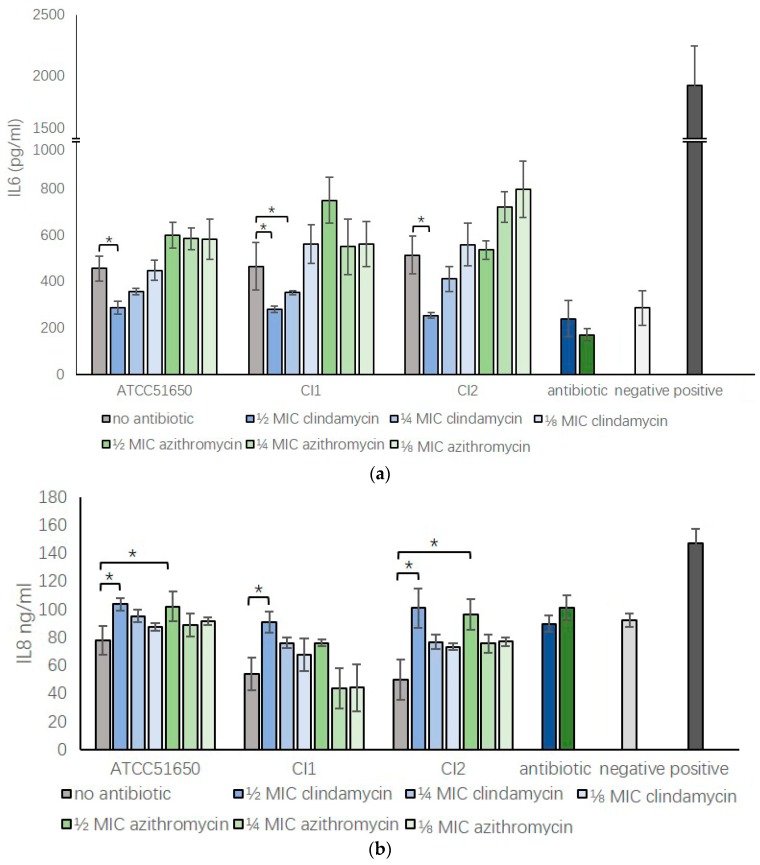
Sub-inhibitory clindamycin and azithromycin can modulate the inflammatory response of HNECs exposed to S. aureus exoproteins. IL6 (**a**) and IL8 (**b**) protein concentration in HNEC culture medium after a 24-h challenge with 5% *S. aureus* exoproteins untreated (no antibiotic, grey bars) or treated with ½, ¼ or ⅛ MIC clindamycin (blue bars) or azithromycin (green bars) for 24 h. Positive control = 10 μg/mL Poly (I:C) LMW; negative control = 5% tryptic soy broth in cell culture medium; Antibiotic control = ½ MIC clindamycin (dark blue) or azithromycin (dark green) in medium. *n* = 3. * *p* < 0.05 one-way ANOVA followed LSD post hoc comparison.

We harvested *S. aureus* ATCC 51650, CI1 and CI2 exoproteins after treatment with ½, ¼, ⅛ MIC clindamycin or azithromycin for 24 h and added exoproteins into HNEC culture media at a concentration of 5%. We used 10 μg/mL Poly I:C (LMW) as a positive control to induce inflammation and 5% bacterial growth medium in PneumaCult^TM^-EX plus medium as negative control. After 24 h culture, IL6 and IL8 concentration were tested using ELISA assays. *S. aureus* ATCC 51650, CI1 and CI2 exoproteins significantly enhanced the IL6 secretion in HNECs compared to negative control (*p* < 0.05). Compared to those, exoproteins harvested from ½ MIC clindamycin treated *S. aureus* significantly reduced the bacterial exoprotein induced IL6 secretion back to background levels for all three isolates (*p* < 0.5). However, ½ MIC azithromycin did not show the same effect (Figure 5a, *p* = 0.99 for ATCC51650, *p* = 0.97 for CI1 and *p* = 0.38 for CI2). In contrast, compared with negative control, *S. aureus* supernatant reduced the basal IL8 secretion of HNECs for all three isolates (*p* < 0.05). ½ MIC clindamycin and azithromycin treatment reversed this reduction to the control level in 3/3 and 2/3 isolates, respectively (*p* < 0.05) (Figure 5b). 

### 3.6. Sub-Inhibitory Clindamycin Treatment of S. aureus Reduced Intracellular Infection Rate of HNECs

**Figure 6:**
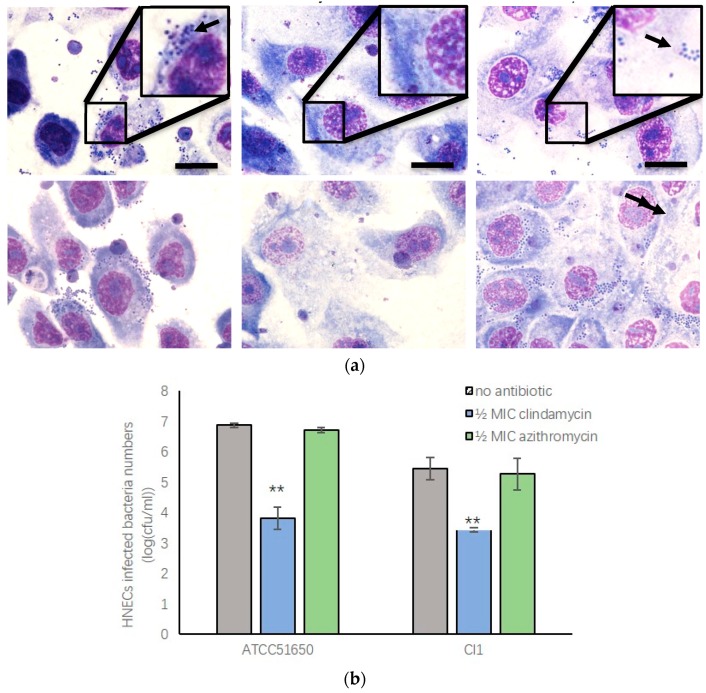
½ MIC clindamycin treatment of *S. aureus* reduced intracellular infection of HNECs. **a**: Giemsa staining of HNECs with different antibiotic treated *S. aureus* ATCC51650 and CI1 showing the presence of intracellular cocci (arrow). **b**: Colony Forming Units (log CFU/mL) of intracellular *S. aureus* ATCC 51560 and CI1 in HNECs. ** *p* < 0.01, one-way ANOVA followed LSD post hoc comparison.

*S. aureus* can invade mammalian cells, which is thought to contribute to the chronic relapsing nature of *S. aureus* infections in the context of CRS [8,26]. To test the effect of sub-inhibitory clindamycin and azithromycin on intracellular infection rate, we first treated *S. aureus* with ½ MIC clindamycin or azithromycin for 24 h before co-culturing the bacteria with HNECs for 3 h. To test the bacterial infection rate, Giemsa staining of HNECs was performed and colony forming units (CFUs) were counted after removal of extracellular bacteria and cell lysis. Giemsa staining showed ATCC 51650 and CI1 positive control cells had infection rates of 49 ± 9.8% and 26.9 ± 3.3%, whilst CI2 had a very low infection rate of 4 ± 0.6%. Cells treated with ½ MIC clindamycin, but not azithromycin, significantly reduced intracellular infection rate of ATCC 51650 and CI1 (Figure 6a and Table 4). There was a significant reduction of CFUs in HNECs infected with ½ MIC clindamycin treated bacteria compared with control and azithromycin treated bacteria (Figure 6b, *p* < 0.01).

### 3.7. ½ MIC Clindamycin Reversed Bacterial Exoprotein Induced Mucosal Barrier Disruption of HNEC-ALI

**Figure 7:**
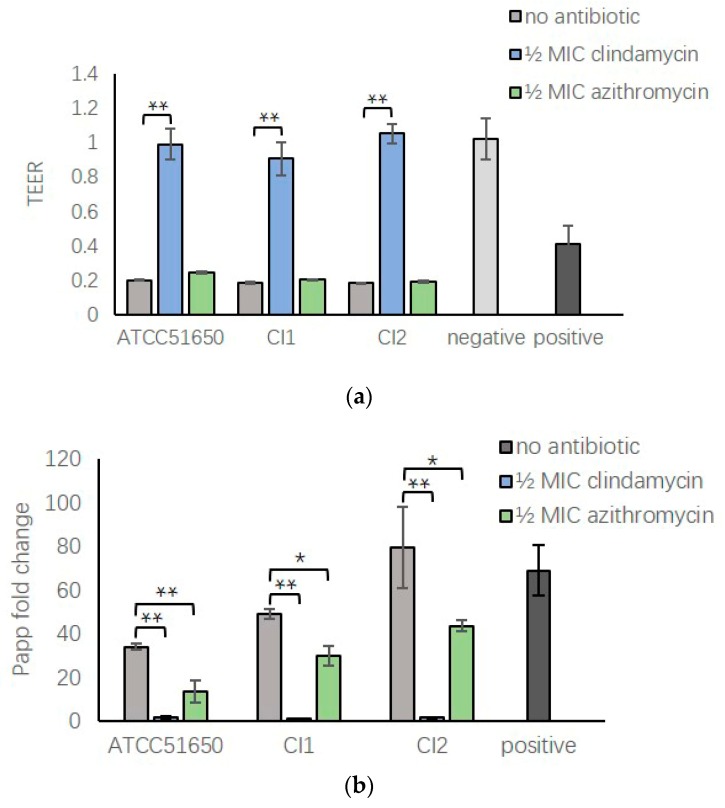

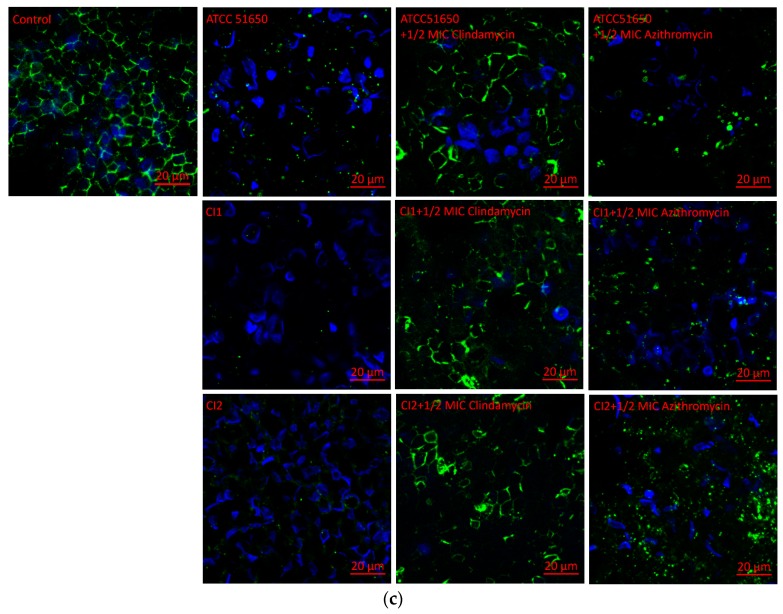
Transepithelial electrical resistance, relative permeability and immunofluorescence staining of *S. aureus* exoproteins treated HNEC-ALI cultures. (**a**,**b**) ½ MIC clindamycin significantly reversed the TEER decrease (**a**) and the increased HNEC-ALI permeability of FITC-dextrans induced by *S. aureus* ATCC 51650, CI1 and CI2 (**b**). (**c**) Immunofluorescence staining of ZO-1 (green) in HNEC-ALI cultures treated with medium (control) or exoproteins from ATCC 51650, CI1 and CI2 left untreated or treated with ½ MIC clindamycin or ½ MIC azithromyxin. DAPI stains nuclei blue. * *p* < 0.05, ** *p* < 0.01. *n* = 3.

Our previous studies found *S. aureus* exoproteins could severely disrupt the mucosal barrier structure and function of HNEC-ALI cultures [27,28,29]. To explore the effect of sub-inhibitory clindamycin and azithromycin on *S. aureus* exoprotein induced barrier dysfunction, *S. aureus* was cultured with and without ½ MIC clindamycin or azithromycin, exoproteins filtered and added to the apical chamber of HNEC-ALI cultures. Non-treated bacterial exoproteins reduced the TEER after 15 min exposure (Figure 7a, *p* < 0.01) and increased the permeability of FITC-dextrans after 30 min (Figure 7b, *p* < 0.01). Compared with the no antibiotic treated group, ½ MIC clindamycin, but not azithromycin, completely reversed the TEER reduction with TEER values similar to negative control. Both antibiotics reduced the increased FITC-dextran permeability of positive control samples, however, the effect was more pronounced with ½ MIC clindamycin treated samples, reducing permeability to background levels (Figure 7b). Immunofluorescence showed that *S. aureus* exoproteins in the presence or absence of ½ MIC azithromycin disrupted ZO-1 immunolocalisation of HNEC-ALI cultures. In contrast, ½ MIC clindamycin treated exoproteins induced no alteration in the localization of ZO-1 compared with negative control (Figure 7c). 

## 4. Discussion

This study showed that sub-inhibitory clindamycin effectively blocked *S. aureus* exoprotein production, thereby inhibiting exoprotein induced toxicity, reversing its detrimental effects on mucosal barrier structure and function and modulating its pro-inflammatory properties. Sub-inhibitory azithromycin had similar effects on affecting these processes—albeit to a lesser extent. Furthermore, clindamycin—but not azithromycin—treated *S. aureus* lost its invasive capacity of primary human nasal epithelial cells. Importantly, whilst up to 250-fold higher concentrations than MIC values were required to kill *S. aureus* biofilms, subinhibitory clindamycin or azithromycin was sufficient to significantly reduce protein production of *S. aureus* biofilms.

Clindamycin, which belongs to the lincosamide class of antibiotics and azithromycin, is a macrolide antibiotic. Unlike antibiotics such as beta-lactam antibiotics, which act on the bacterial cell wall, both clindamycin and azithromycin have a similar mechanism of action and inhibit bacterial protein synthesis by binding to the 50S subunit of the bacterial rRNA [20,30]. As expected, dose-dependent reductions in exoprotein production were seen for both antibiotics—even at concentrations as low as ⅛ MIC values. However, differences between both antibiotics were observed where exoprotein quantities in the presence of subinhibitory clindamycin were consistently lower than in the presence of azithromycin. These differences could account for the variability seen in the attenuation of exoprotein-induced cell toxicity and inflammation in the presence of both antibiotics. Interestingly, whilst the effect of subinhibitory azithromycin on attenuation of exoprotein dependent inflammation was evident for IL8, no differences were observed in HNEC IL6 production between exoproteins treated or untreated with subinhibitory azithromycin. Furthermore, whereas subinhibitory clindamycin completely reversed exoprotein induced barrier disruption and manifestly reduced intracellular infection rate of all three isolates, subinhibitory azithromycin had no effect on these processes. It is hypothesized that apart from the overall reduction in the amount of exoproteins that is produced in the presence of those antibiotics, qualitative differences in expression of proteins might occur where some specific proteins might be induced or suppressed as well. This phenomenon has been demonstrated for clindamycin where changes at transcriptional and protein level are observed, resulting in an overall suppression of exoprotein production, except for an induction of expression of specific proteins such as coagulase and fibronectin binding protein B [20]. A more detailed gene expression and proteomics analysis would be required to define which genes and proteins are induced or repressed by both antibiotics and how that might affect invasiveness, inflammation and mucosal barrier disruption.

*S. aureus* is one of the most commonly identified bacteria in the sinonasal cavities of CRS patients and *S. aureus* mucosal biofilms are often found in those patients with more severe recalcitrant disease [4,7,31,32,33]. In line with our findings of up to 250-fold MIC concentrations of clindamycin and azithromycin needed to kill *S. aureus* biofilms, *S. aureus* biofilms are known to be significantly less sensitive to bacterial killing by antibiotics than their planktonic counterparts. Regardless, our study indicates that treatment with low concentrations of ½ MIC of clindamycin and azithromycin was sufficient to significantly reduce the bacterial exoprotein production in *S. aureus* biofilms. This has important clinical implications and indicates that MIC level antibiotic concentrations, used to treat acute infectious exacerbations, might reduce *S. aureus* biofilm exoprotein induced inflammation and toxicity, in addition to reducing the bacterial burden by killing planktonic cells. Further in vivo experiments would be required to assess this hypothesis. 

This study showed that *S. aureus* exoproteins of all three isolates exerted significant cytotoxic effects when applied to HNECs. In fact, 10% *S. aureus* exoproteins induced similar toxicity as 10% Triton X-100, one of the most widely used non-ionic surfactants for lysing cells. Numerous virulence factors are secreted by *S. aureus* that could contribute to this toxicity, including cytolysins and proteases, which are known to cause cell damage [34,35]. These same factors could also be the cause of the mitigation of inflammation and the acute detrimental effects on the mucosal barrier function. It is postulated that exoproteins secreted by the bacteria might significantly contribute to the inflammation observed in the context of *S. aureus* infection and that low concentrations of azithromycin and clindamycin might reduce exoprotein induced inflammation in vivo as well. Low concentrations of macrolides are often used as anti-inflammatory agents for the management of a number of severe chronic inflammatory diseases of the airways [15,16,17,18]. In line with our findings where such low concentrations of antibiotics did not affect *S. aureus* growth, bacteria remain present in the sputum of patients—despite the reduction of inflammation upon long-term treatment with subinhibitory macrolides [15,16,17,18]. In view of our findings, it is reasonable to hypothesise that at least part of the anti-inflammatory effect of subinhibitory macrolide antibiotics might be secondary to reduced exoprotein production. 

This study showed reciprocal effects on inflammatory cytokine production with increased and decreased IL6 and IL8 production by HNECs upon application of *S. aureus* exoproteins. Different proteins within *S. aureus* supernatants could influence the inflammatory process and resulting inflammatory cytokine production. IL6 and IL8 protein levels were increased in mucosal explants exposed to *S. aureus* biofilms [36], whilst IL6 can be degraded by specific *S. aureus* proteases as well [29]. Similar to this study, Tajima et al. showed that *S. aureus* β-hemolysin inhibited the human umbilical vein endothelial cell IL8 production [37]. Taken together, these findings indicate a balancing act where bacterial exoproteins might be responsible for inducing inflammation, whilst at the same time reducing specific inflammatory cytokine levels as well, thereby preventing the recruitment of specific immune cells that would be needed to kill the bacteria. Normalizing cytokine levels with the use of subinhibitory clindamycin might therefore reduce inflammation and at the same time help the immune system to eliminate the infection.

## 5. Conclusions

This study indicates that *S. aureus* exoproteins incite severe cytotoxicity, disrupt the mucosal barrier structure and function and attenuate inflammation. These processes are blocked by subinhibitory clindamycin and to a lesser extent azithromycin—indicating the potential use of these antibiotics to reduce the inflammatory cascade in severe *S. aureus* infections. Additionally, clindamycin could reduce *S. aureus* invasiveness of HNEC, further reducing the potential of this organism to cause chronic relapsing infections.

## Figures and Tables

**Table 1:** *S. aureus* minimal inhibitory concentration for clindamycin and azithromycin (μg/mL) for ATCC51650, Clinical Isolate 1 (CI1) and 2 (CI2).

Antibiotic	ATCC51650	CI1	CI2
Clindamycin (μg/mL)	0.2	0.2	0.2
Azithromycin (μg/mL)	2	2	2

**Table 2:** Gray scale analysis of the sodium dodecyl sulphate polyacrylamide gel electrophoresis (SDS-PAGE) with Image J. (compared with no antibiotic treatment, * *p* < 0.05, one-way analysis of variance (ANOVA) followed by Least Significant Difference (LSD) post hoc comparison.). CI= clinical isolate, MIC = minimum inhibitory concentration.

Antibiotic	ATCC51650	CI1	CI2
No antibiotic	41.31 ± 2.36	37.91 ± 2.48	10.74 ± 2.60
½ MIC clindamycin	12.22 ± 0.58 *	9.18 ± 0.53 *	1.51 ± 1.56 *
¼ MIC clindamycin	18.31 ± 0.90*	11.79 ± 1.08 *	3.96 ± 1.10 *
⅛ MIC clindamycin	21.45 ± 1.82 *	16.99 ± 1.23 *	8.51 ± 1.85
½ MIC azithromycin	18.48 ± 0.70 *	25.06 ± 1.89 *	3.94 ± 2.28 *
¼ MIC azithromycin	23.82 ± 2.27 *	31.03 ± 3.68 *	9.46 ± 1.94
⅛ MIC azithromycin	28.66 ± 0.65 *	32.97 ± 2.14	10.46 ± 1.99

**Table 3:** Gray scale analysis of the SDS-page of 48-h biofilms with Image J. (compared with no antibiotic treatment, * *p* < 0.05, one-way ANOVA followed by LSD post hoc comparison).

Antibiotic	ATCC51650	CI1	CI2
No antibiotic	11.54 ± 1.85	38.85 ± 1.90	15.60 ± 1.04
1 MIC clindamycin	3.80 ± 0.43 *	1.57 ± 0.12 *	1.79 ± 0.49 *
½ MIC clindamycin	10.27 ± 1.09	7.51 ± 0.68 *	1.57 ± 0.88 *
1 MIC azithromycin	7.20 ± 0.49 *	6.35 ± 0.68 *	1.86 ± 0.58 *
½ MIC azithromycin	10.15 ± 2.09	7.43 ± 0.64 *	7.22 ± 2.07 *

**Table 4:** *S. aureus* intracellular infection rate. Cells were Giemsa stained and % of HNECs infected by *S. aureus* quantified and the average of three independent infection rates expressed as mean ± SEM. Compared with no antibiotic treatment, ½ MIC clindamycin significantly reduced intracellular infection rate of ATCC 51650 and CI1 (** *p* < 0.01).

Antibiotic	ATCC51650	CI1	CI2
No antibiotic	49 ± 9.8%	26.9 ± 3.3%	4 ± 0.6%
½ MIC Clindamycin	6.3 ± 0.76% **	5.6 ± 3.8% **	2.9 ± 2.6%
½ MIC Azithromycin	42.8 ± 10.7%	27.4 ± 19.7%	3.6 ± 2.3%
